# Supplementary material for: Stable structures or PABP1 loading protects cellular and viral RNAs against ISG20-mediated decay
Source: Life Sci Alliance. 2024 Feb 28;7(5):e202302233. doi: 10.26508/lsa.202302233 (PMC10902665; doi:10.26508/lsa.202302233)
Supplement: Supplementary file 1 [file LSA-2023-02233_SdataF1.1_F2.1_F3.1_F4.1_F5.1_F6.1_F7.1_FS3.1_FS4.1.pdf]

Figure in manuscript

Louvat et al., 2023, Figure 1

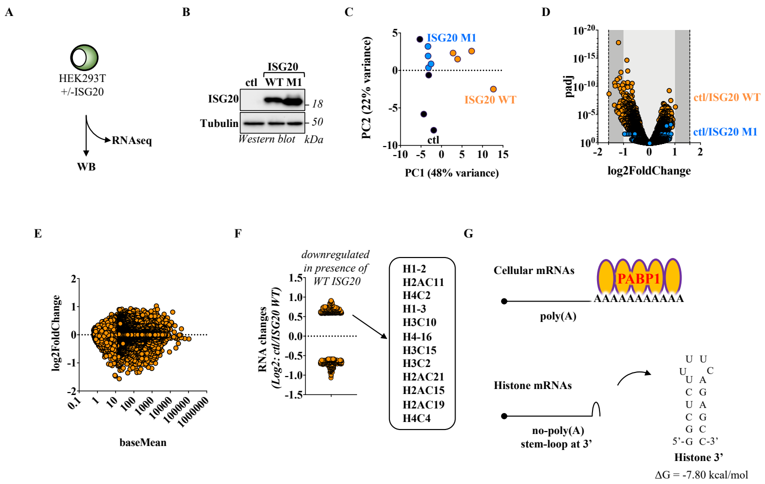

Corresponding blots

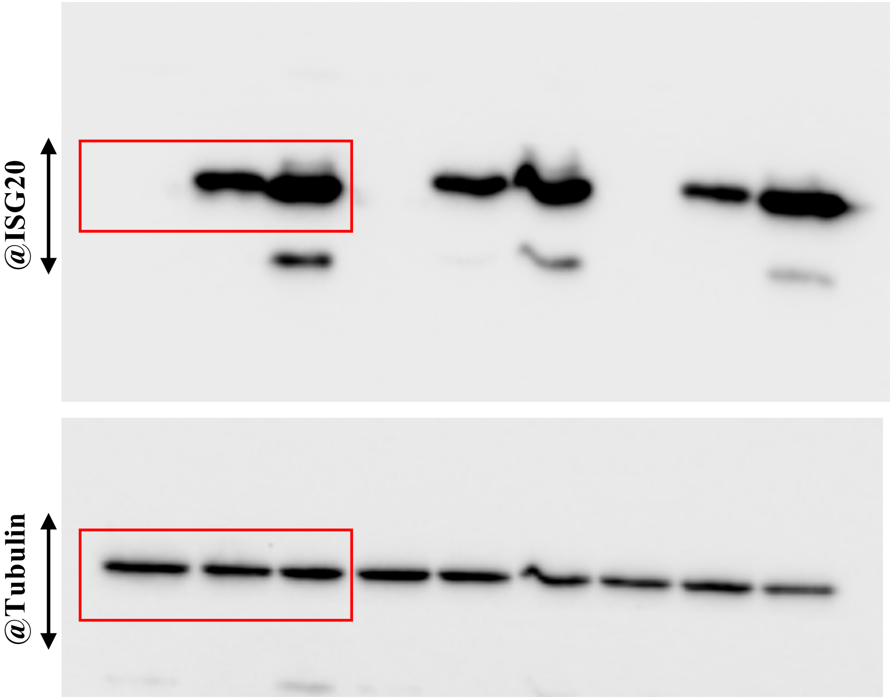

non circled=unrelated samples

Figure in manuscript

Louvat et al., 2023, Figure 2

Corresponding blots

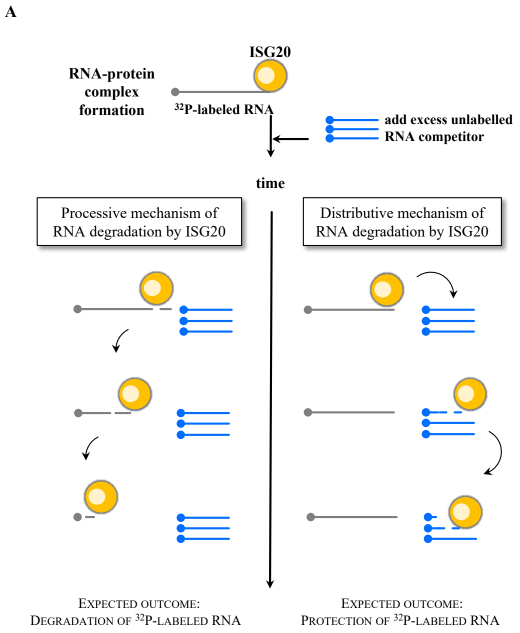

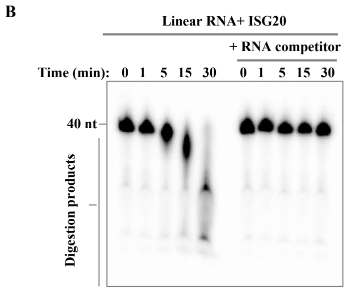

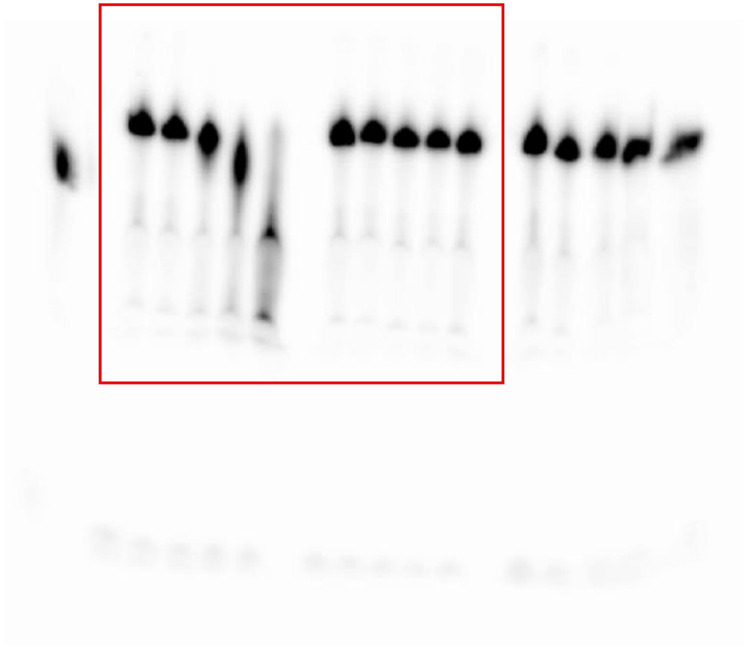

non circled=unrelated samples

Figure in manuscript

Louvat et al., 2023, Figure 3

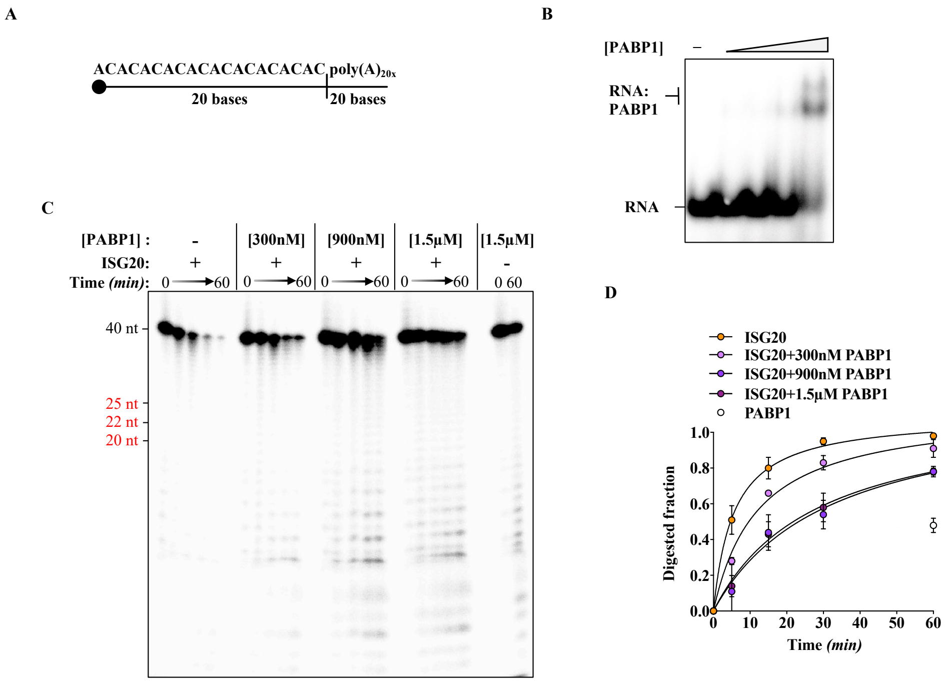

Corresponding blots

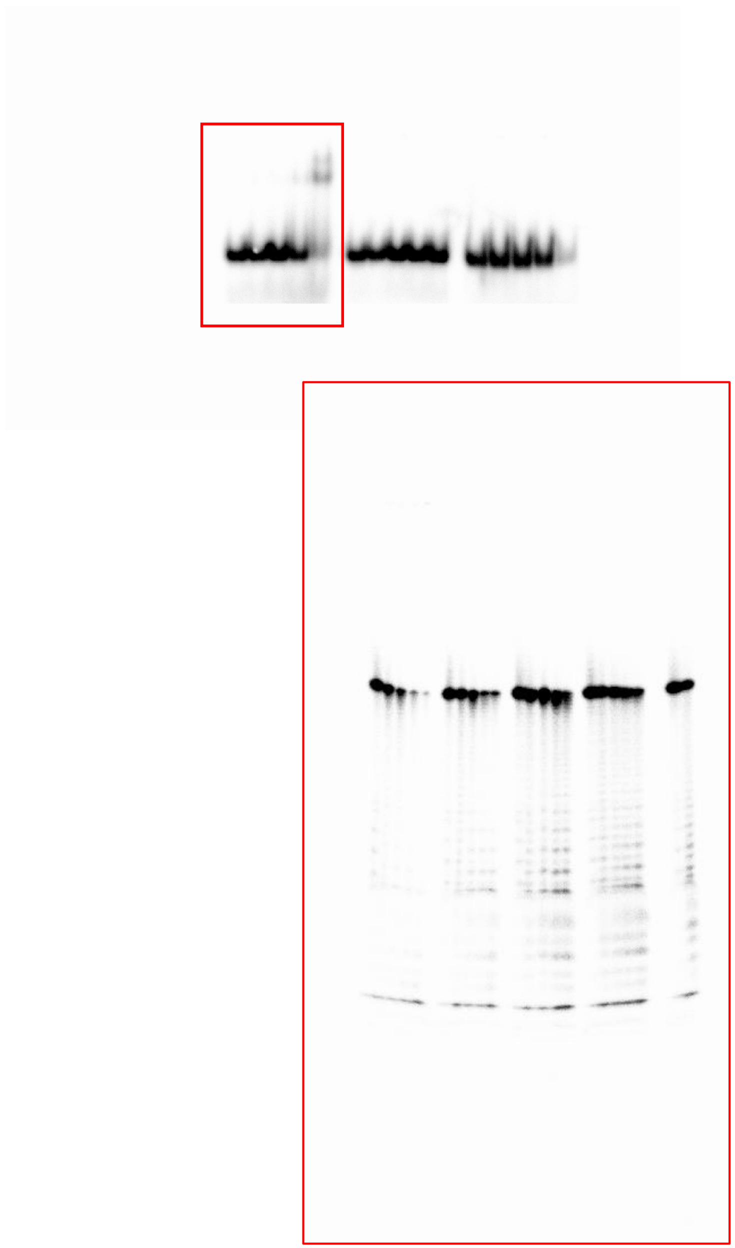

non circled=unrelated samples

Figure in manuscript

Louvat et al., 2023, Figure 4

Corresponding blots

A

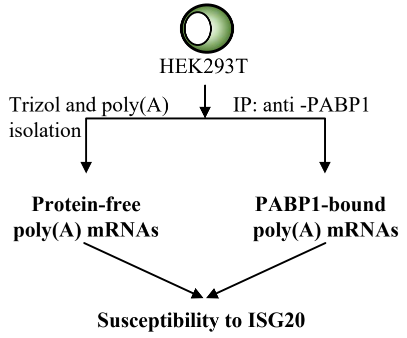

B

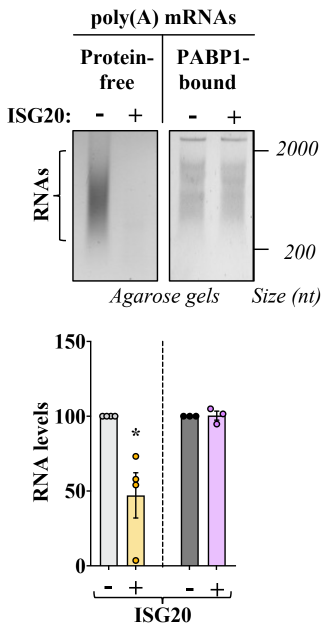

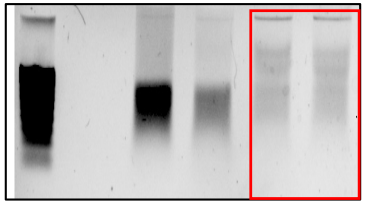

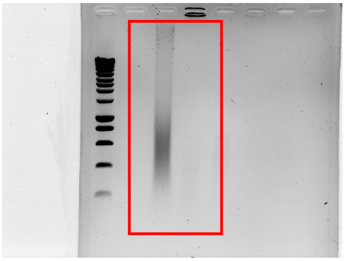

non circled=unrelated samples

Figure in manuscript

Louvat et al., 2023, Figure 5

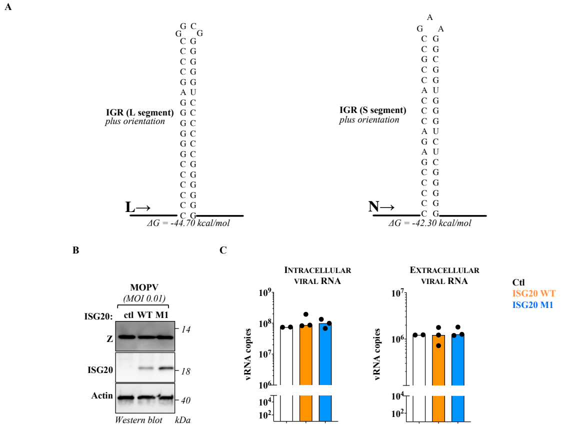

Corresponding blots

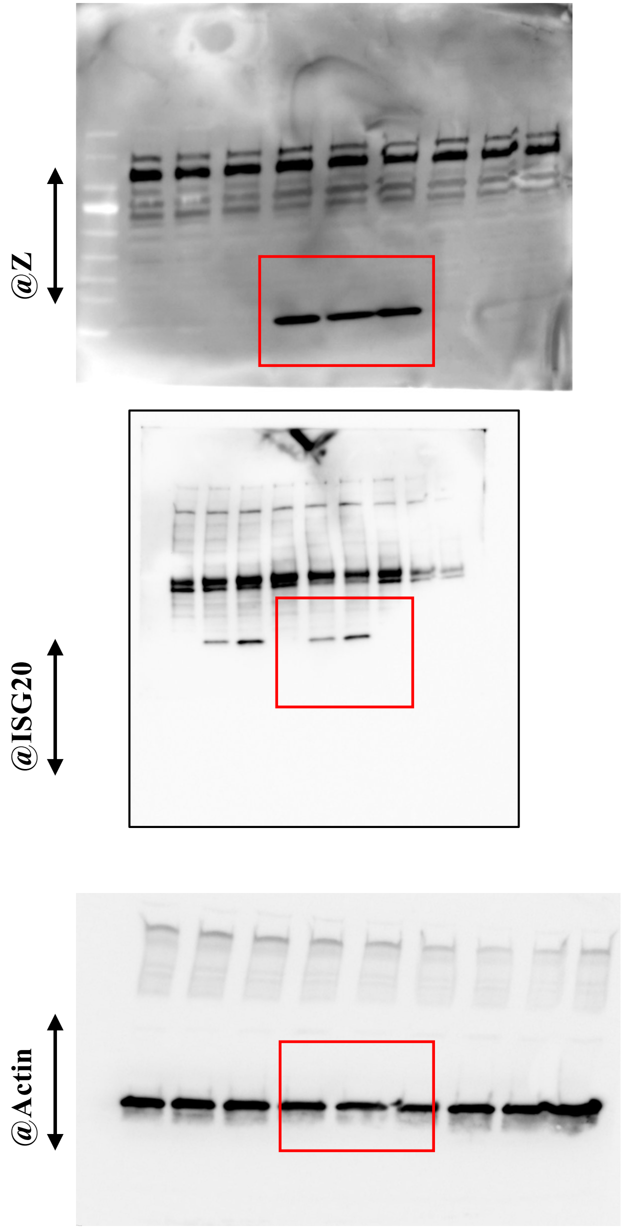

non circled=unrelated samples

Figure in manuscript

Louvat et al., 2023, Figure 6

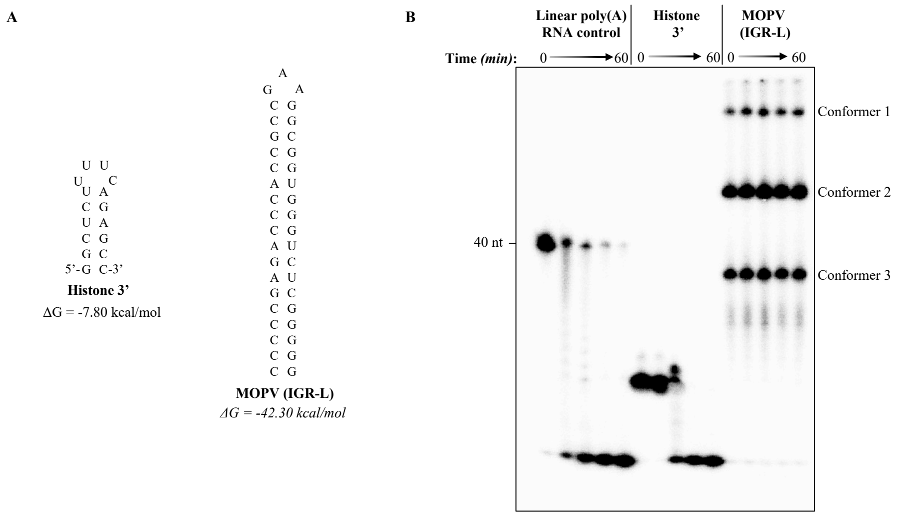

Corresponding blots

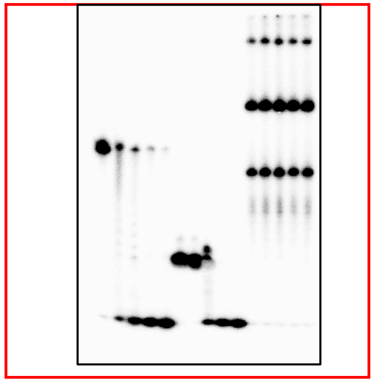

non circled=unrelated samples

Figure in manuscript

Louvat et al., 2023, Figure 7

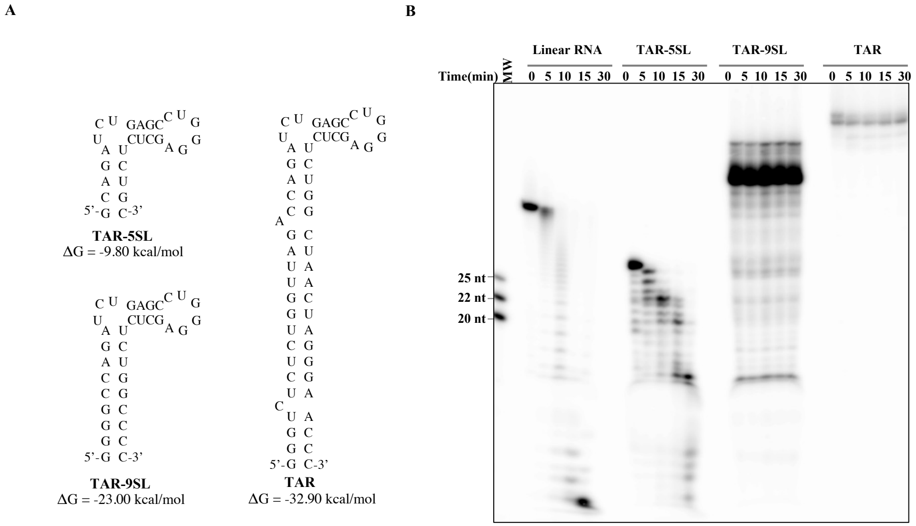

Corresponding blots

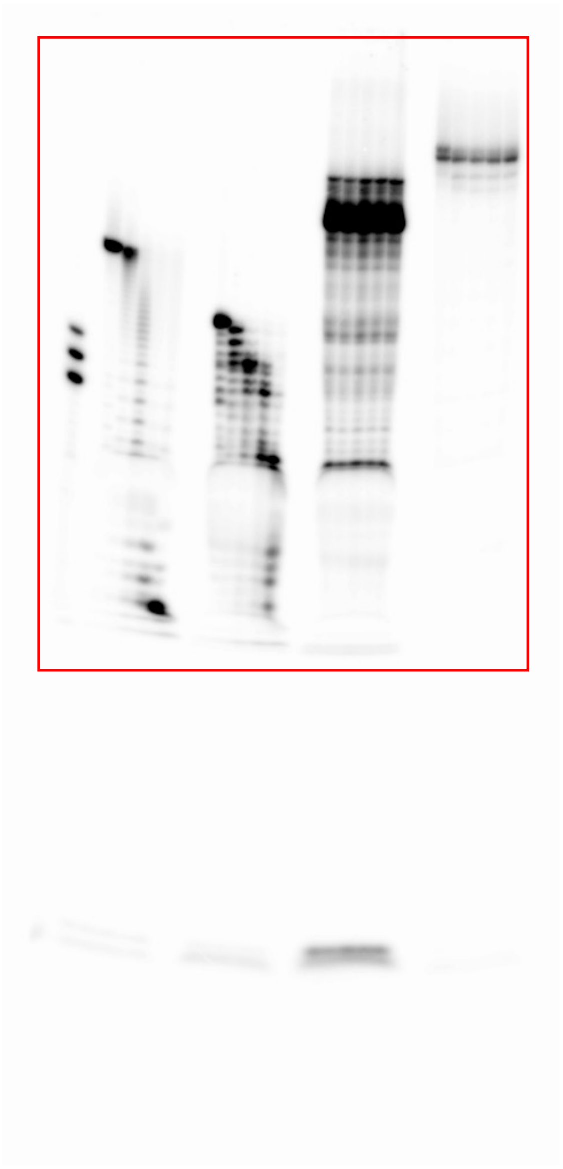

non circled=unrelated samples

Figure in manuscript

Louvat et al., 2023,
Supplementary Figure 3

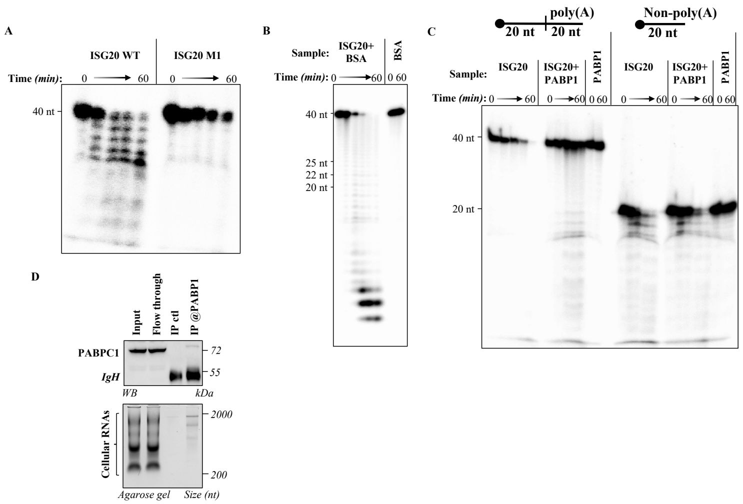

Corresponding blots

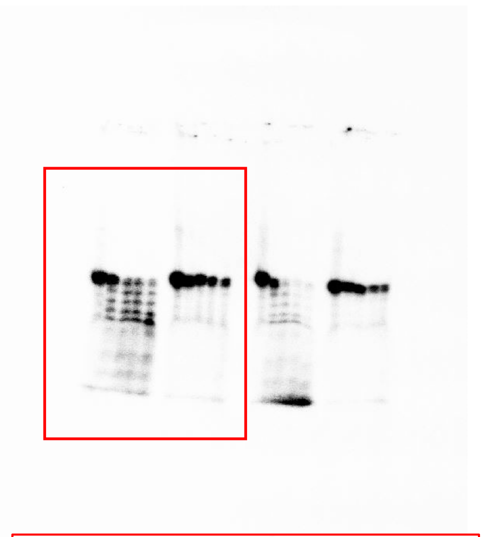

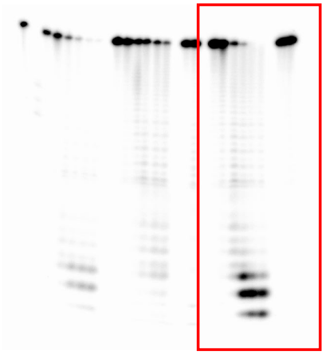

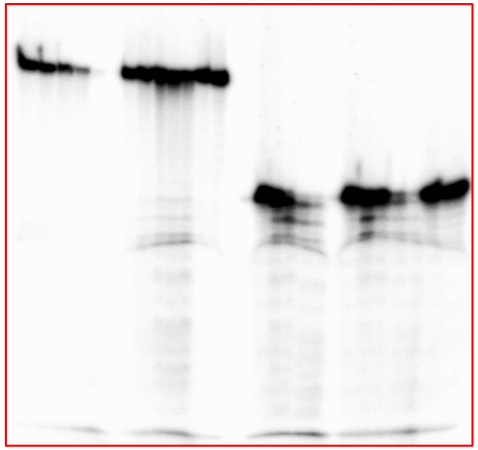

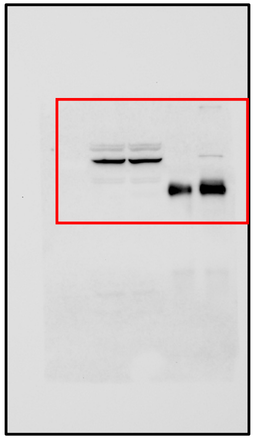

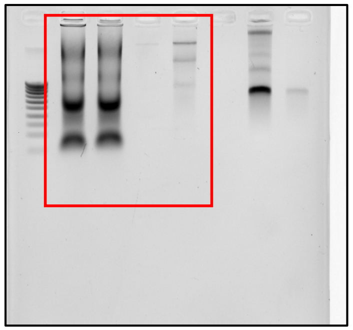

non circled=unrelated samples

Figure in manuscript

Louvat et al., 2023,
Supplementary Figure 4

Corresponding blots

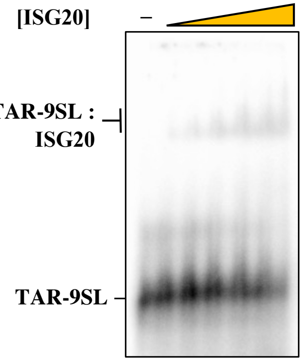

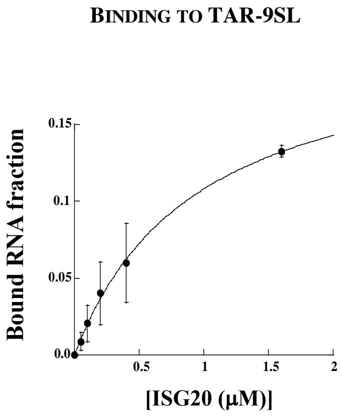

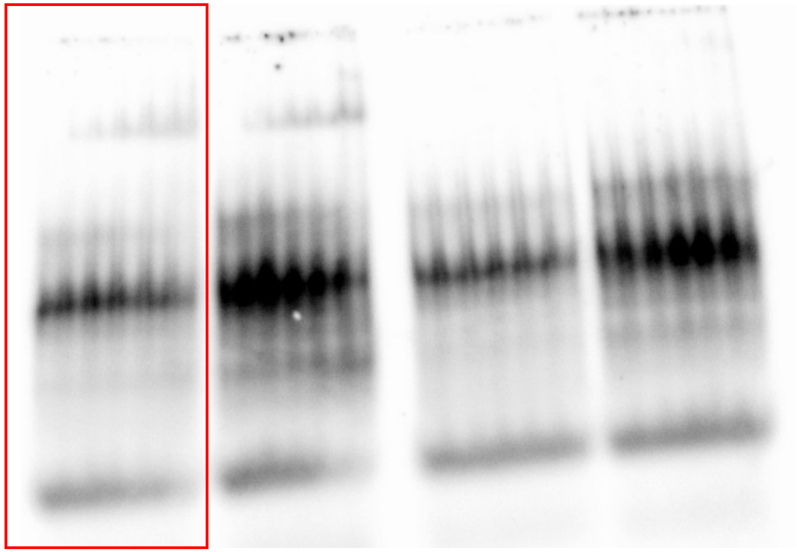

non circled=unrelated samples
