# Supplementary material for: Stable structures or PABP1 loading protects cellular and viral RNAs against ISG20-mediated decay
Source: Life Sci Alliance. 2024 Feb 28;7(5):e202302233. doi: 10.26508/lsa.202302233 (PMC10902665; doi:10.26508/lsa.202302233)
Supplement: Supplementary file 3 [file LSA-2023-02233_TableS1.docx]

**Supplementary Table 1.**

|  | **Ctl vs WT ISG20** | | **Ctl vs M1 ISG20** | |
| --- | --- | --- | --- | --- |
| **gene_name** | **log2FoldChange** | **padj** | **log2FoldChange** | **padj** |
| ISG20 | -7,661306658 | 0 | -7.05877453162163 | 0 |
| CCDC81 | -1,06796482 | 0,00010116 | -0.190895238348612 | NA |
| ADAM20 | -1,012088193 | 0,00011902 | -0.255066808944163 | 0.70047253686956 |
| ZNF700 | -0,985266391 | 0,00021516 | -0.409034770372517 | 0.52866142971364 |
| LRIT3 | -0,923226028 | 0,00074381 | -0.188616794120772 | NA |
| DNAH17 | -0,922708087 | 0,00031548 | 0.194224854405005 | 0.761290236503316 |
| SCG2 | -0,908652187 | 0,0007879 | 0.26261678347922 | NA |
| LINC02641 | -0,893835882 | 0,00096433 | 0.190452208211435 | 0.775513275481614 |
| H2BC19P | -0,889899619 | 0,00067713 | 0.00970897954402269 | 0.990985265613943 |
| PRECSIT | -0,887714754 | 0,00128732 | 0.0857499771007512 | NA |
| RIMS2 | -0,879458104 | 0,00111682 | 0.312030784123918 | 0.630751945415625 |
| ZNF441 | -0,867950104 | 0,00010455 | -0.0294454803557095 | 0.965418374907633 |
| ZNF221 | -0,863574125 | 0,00025294 | -0.0146313873021315 | 0.986114969632313 |
| ALDH8A1 | -0,85921976 | 0,00078364 | -0.258065764517868 | 0.680418040657311 |
| MAFG | -0,856245977 | 0,00094841 | 0.0705618833596952 | 0.919628250154625 |
| TXNIP | -0,848882283 | 0,0028156 | -0.156102157313806 | 0.817727051192506 |
| PAX5 | -0,844308121 | 0,00261933 | 0.0884896861608723 | NA |
| VCPKMT | -0,843381387 | 0,00013466 | -0.0679085609387154 | 0.911212382028329 |
| LRRTM2 | -0,829876647 | 0,003263 | 0.197619274142745 | 0.770145332677736 |
| ZFP37 | -0,821234826 | 0,001314 | -0.097986123919935 | 0.884836337261322 |
| YY2 | -0,820820606 | 0,00091883 | 0.134923822577304 | 0.825864924370673 |
| ZNF44 | -0,82053883 | 0,00022606 | -0.17658193954764 | 0.755678401412281 |
| GAS2L3 | -0,81890102 | 0,00022324 | -0.323667657281818 | 0.542471401406355 |
| CELSR3 | -0,812910506 | 0,00037984 | 0.454337061794093 | 0.412595764957876 |
| ZNF510 | -0,808393053 | 0,00012882 | -0.370833737236559 | 0.476438741954515 |
| ZNF222 | -0,805453453 | 0,00138436 | -0.360552265396217 | 0.546028796569388 |
| PI15 | -0,800908918 | 0,00022818 | -0.266167878560138 | 0.620367075161585 |
| UNC79 | -0,794072015 | 0,00493001 | 0.077039997086672 | 0.917451478220486 |
| MAGEB2 | -0,784270425 | 0,00439579 | 0.0495415323124501 | 0.949452837327508 |
| ZNF586 | -0,781574039 | 0,00027298 | -0.0206679391980055 | 0.974432189083573 |
| EPC1 | -0,768979392 | 0,00019713 | 0.0261388376271488 | 0.966148557294968 |
| ZNF225 | -0,76513745 | 0,00010455 | 0.0198531889598106 | 0.974354983182563 |
| ZNF274 | -0,763359657 | 0,00017897 | 0.162088010918277 | 0.754139098311339 |
| ZNF814 | -0,758769367 | 0,00269131 | -0.06107239588538 | 0.930871538856666 |
| CPEB4 | -0,758431846 | 0,00011585 | 0.0113698400541168 | 0.986662271086382 |
| ZNF419 | -0,756368972 | 0,00022818 | 0.0148728972358003 | 0.981979722419876 |
| PAN3-AS1 | -0,748992512 | 0,00952613 | -0.0632788347125583 | NA |
| CREBRF | -0,745729482 | 0,00165766 | -0.164930339016603 | 0.773535565764064 |
| PTHLH | -0,745231721 | 0,00971569 | -0.151751081821399 | NA |
| ZNF35 | -0,74161754 | 0,00041649 | -0.112755742561614 | 0.828536431513732 |
| DCUN1D3 | -0,741259937 | 0,00016916 | -0.148713435576518 | 0.766283264049794 |
| ARRDC4 | -0,739243256 | 0,00036789 | -0.385763970995456 | 0.447983429151844 |
| ZNF66 | -0,737611853 | 0,00440501 | -0.0106248318969817 | 0.990160210870583 |
| DDIT3 | -0,735922252 | 0,00342624 | -0.0461021056045134 | 0.949175299620477 |
| TTLL7-IT1 | -0,733075325 | 0,00946053 | -0.324670248457351 | NA |
| ZNF112 | -0,732238717 | 0,00086364 | -0.0899903345286747 | 0.879140263375176 |
| SYT1 | -0,730006269 | 0,00027298 | -0.0697090945492841 | 0.898627347466607 |
| ZNF684 | -0,727446613 | 0,00087971 | 0.0344242763010522 | 0.957733689170877 |
| ZNF211 | -0,725818531 | 0,00022845 | 0.170829662432121 | 0.734332674317012 |
| SEMA3C | -0,720947421 | 0,00033404 | -0.248668928808947 | 0.614426457410965 |
| MYLK3 | -0,718240695 | 0,01176282 | 0.0625837137408755 | 0.936364654306675 |
| TECTA | -0,717446884 | 0,00452726 | -0.0585663460504601 | 0.934043816615612 |
| ZNF182 | -0,714515707 | 0,00018097 | -0.126550072870014 | 0.791102781925862 |
| CHIC1 | -0,710920291 | 0,00082936 | -0.127355392462986 | 0.80513507336883 |
| ZNF568 | -0,710449715 | 0,0011154 | -0.0908354819912892 | 0.876124589398383 |
| H3C6 | -0,709956946 | 0,006016 | -0.0393000286434212 | 0.956926638808855 |
| ZNF557 | -0,707929988 | 0,00017488 | 0.0784944866771092 | 0.87793571978977 |
| KLF2 | -0,707098842 | 0,01501603 | -0.00810017553161338 | NA |
| ZNF10 | -0,706951004 | 0,0007712 | -0.0591848321345346 | 0.918006651608884 |
| GCC2 | -0,706313117 | 0,00025879 | -0.17841893528437 | 0.711422360713909 |
| ERVK3-1 | -0,705614492 | 0,00060871 | -0.272596127008919 | 0.582558442417588 |
| C2orf49-DT | -0,703139905 | 0,0007712 | -0.0804964382275309 | 0.83102737348859 |
| XDH | -0,702627982 | 0,00293518 | 0.259614306818848 | 0.649676554842228 |
| ZNF184 | -0,701181865 | 0,00013073 | -0.0929214955058795 | 0.846884109731693 |
| SHLD3 | -0,698494165 | 0,01157995 | -0.0783259584661479 | 0.912629715961281 |
| UBOX5 | -0,697537803 | 0,00244133 | 0.066836491534377 | 0.91373035199419 |
| PTPRG-AS1 | -0,693197605 | 0,00012758 | -0.067485297329047 | 0.892581625574937 |
| PYGO1 | -0,692966647 | 0,00166671 | -0.212458439520778 | 0.688897679020411 |
| ZNF773 | -0,692120367 | 0,00448421 | 0.217163062007156 | 0.714096484384976 |
| MTF1 | -0,690405271 | 0,00060496 | 0.132520754277171 | 0.787269269420425 |
| ADAM32 | -0,687079814 | 0,01898467 | -0.433321818266214 | 0.514948163136529 |
| DUSP8 | -0,686893983 | 0,01487869 | 0.324348539716734 | 0.619605742211831 |
| EMBP1 | -0,686324425 | 0,00942167 | -0.297637929449265 | 0.630751945415625 |
| SRFBP1 | -0,685688055 | 0,00012493 | -0.0565907334064566 | 0.907236811634517 |
| LINC02864 | -0,683977295 | 0,0009501 | -0.249701131930833 | 0.619605742211831 |
| METTL15P1 | -0,683029368 | 0,01898467 | -0.118634618162083 | NA |
| BAALC-AS1 | -0,682707145 | 0,01252622 | 0.147536906314506 | 0.818875587381845 |
| ZXDA | -0,681724927 | 0,00286789 | -0.156377756354323 | 0.777710310862195 |
| ZNF681 | -0,681036887 | 0,0202582 | -0.159914211950607 | NA |
| ZNF267 | -0,680621847 | 0,00013073 | -0.212682742127789 | 0.625857136528475 |
| TNRC18P1 | -0,679347483 | 0,01551307 | 0.0256621758771183 | NA |
| MGAM2 | -0,678795015 | 0,01954822 | 0.301609826344736 | NA |
| PPP1R15A | -0,678669598 | 0,01933838 | 0.176238256728226 | 0.7923388973596 |
| CASP8AP2 | -0,678357397 | 0,00054934 | -0.0796483336430939 | 0.878530875432954 |
| ZNF649 | -0,676771044 | 0,00016014 | -0.0308355987880772 | 0.954528320048073 |
| RANBP6 | -0,67675369 | 0,00048224 | -0.149719739493685 | 0.758104129781368 |
| ERI2 | -0,676217437 | 0,00022405 | -0.0440314519072923 | 0.931116766490199 |
| ZNF764 | -0,675654964 | 0,00237576 | 0.296249279798912 | 0.574570114296114 |
| NTAN1P2 | -0,672833705 | 0,02211484 | -0.0385618088842058 | NA |
| ZNF254 | -0,671515282 | 0,00056682 | -0.157103381503131 | 0.747765785719127 |
| PGBD4 | -0,670992126 | 0,00485148 | -0.222278682828417 | 0.699691729336992 |
| RORA | -0,668298149 | 0,00029051 | -0.385350812412534 | 0.369781661436704 |
| OR2B6 | -0,667430142 | 0,00388316 | 0.0238041716619617 | NA |
| ZNF331 | -0,667352126 | 0,0078696 | -0.001427074307925 | 0.999099250746181 |
| NECAP1 | -0,666228012 | 0,00127475 | -0.230558082765278 | 0.640405924814128 |
| TMEM79 | -0,665910696 | 0,01056222 | 0.394491208648667 | 0.518348557206828 |
| FAM83G | -0,66478843 | 0,00326869 | 0.0732540627595632 | 0.901911166522359 |
| ZNF480 | -0,664783385 | 0,00221176 | 0.0214177139286935 | 0.973738274520173 |
| ZNF28 | -0,664460005 | 0,00185356 | -0.159968477819131 | 0.762093563924857 |
| ADCY10 | -0,664172711 | 0,02291782 | -0.241591123936813 | NA |
| ZNF502 | -0,663715291 | 0,0112561 | 0.181880857260795 | 0.771831235170964 |
| LINS1 | -0,662100092 | 0,0015713 | -0.0390915827214005 | 0.948296634651027 |
| PBLD | -0,66195426 | 0,00561381 | 0.00864680284818929 | 0.990985265613943 |
| FAM160B1 | -0,661858122 | 0,00042937 | -0.300649028712499 | 0.502510065074613 |
| DNAH7 | -0,661077043 | 0,00561381 | -0.01466480706926 | 0.984889584504117 |
| SLCO6A1 | -0,658720827 | 0,01474635 | -0.0481270282713149 | 0.949175299620477 |
| ANKRD42 | -0,658090366 | 0,00011902 | 0.0611745096960214 | 0.896791896939509 |
| ATG4A | -0,657874894 | 0,00153428 | -0.144433771528537 | 0.77646612295783 |
| LINC00882 | -0,657838223 | 0,0191092 | 0.232895396311013 | 0.728628985755126 |
| PTPN21 | -0,657243814 | 0,00020941 | 0.151505908376356 | 0.738559833157416 |
| NSFP1 | -0,656713894 | 0,01696891 | -0.0212742914604644 | 0.979449369404031 |
| ZNF644 | -0,655692473 | 0,00116674 | -0.164000474616181 | 0.741417299826115 |
| EFL1P1 | -0,655145749 | 0,02012983 | -0.246307212378815 | 0.711422360713909 |
| PSMC3IP | -0,653370631 | 0,02126024 | 0.255322676604974 | 0.698085752412514 |
| DYNLT2 | -0,651254398 | 0,0199352 | -0.223592781369438 | 0.735213630586126 |
| JUN | -0,650985435 | 0,00136501 | 0.134314538438573 | 0.783502878427628 |
| ZNF513 | -0,650651665 | 0,00412886 | -0.02675331738374 | 0.967312188621558 |
| BLZF1 | -0,650271002 | 0,00019813 | -0.0550621516759602 | 0.907244694005636 |
| SPOPL | -0,649962523 | 0,00196574 | -0.131494420810416 | 0.794414506880466 |
| ZNF350 | -0,648856712 | 0,00842977 | -0.0942044884555905 | 0.883348440499811 |
| HELQ | -0,648159859 | 0,00084728 | 0.00808278046495712 | 0.990160210870583 |
| ZNF461 | -0,645483306 | 0,00422712 | 0.0771499913869308 | 0.897728007051635 |
| ZNF583 | -0,645260292 | 0,00361925 | 0.121673372465943 | 0.821704128291882 |
| SLC7A11 | -0,644780878 | 0,00057016 | -0.047139013336724 | 0.928135716638158 |
| EEF1A1P19 | -0,64210518 | 0,02693737 | -0.179332558744339 | NA |
| RICTOR | -0,641138477 | 0,00091076 | -0.225090242196742 | 0.626283886439149 |
| GTF2H2C | -0,638948167 | 0,00188401 | -0.207069532181602 | 0.677966552740841 |
| KDM6A | -0,63779359 | 0,00010341 | -0.209532926440372 | 0.605416958494931 |
| ZNF107 | -0,637642047 | 0,00092525 | -0.150000184967734 | 0.753775513735004 |
| ZNF75A | -0,634951656 | 0,0002052 | -0.0165319850210404 | 0.974432189083573 |
| SRRM5 | -0,634560005 | 0,03302008 | -0.0110548308461313 | NA |
| ZBTB6 | -0,633356653 | 0,00044273 | 0.028928008002743 | 0.956059004511808 |
| MTATP6P19 | -0,632447235 | 0,03040025 | -0.344210943058047 | NA |
| MIR924HG | -0,631964109 | 0,00743962 | -0.219597953978614 | 0.693493568925943 |
| CHROMR | -0,631783723 | 0,00025714 | -0.258036710718149 | 0.537675917098223 |
| ANKRD20A4P | -0,631519943 | 0,03394908 | -0.198462630651147 | NA |
| OVGP1 | -0,63071119 | 0,01980639 | 0.0223494014936398 | 0.97688907482983 |
| ZRANB2 | -0,628809701 | 0,0004889 | -0.122016112292307 | 0.780906314818032 |
| CENPQ | -0,627878592 | 0,00153006 | -0.15195766742976 | 0.758104129781368 |
| BTG3 | -0,625541368 | 0,00115771 | 0.118095690008862 | 0.800751550741699 |
| RBM4 | -0,625507367 | 0,00324417 | -0.274973198904197 | 0.582558442417588 |
| FGD4 | -0,625364144 | 0,00407145 | -0.0876471194335758 | 0.878018801375363 |
| LINC02739 | -0,624766343 | 0,03617951 | -0.0626698539151422 | NA |
| CCDC173 | -0,624721154 | 0,03443693 | 0.171970378313903 | 0.797780403961755 |
| ZNF611 | -0,624614393 | 0,00056477 | -0.323956269201508 | 0.463158206151584 |
| TTPAL | -0,624611807 | 0,00097773 | 0.169384852187135 | 0.718902304120807 |
| H1-0 | -0,622699191 | 0,03392795 | 0.372263512490844 | 0.570574206928719 |
| ZNF879 | -0,620672644 | 0,01363591 | -0.155361642197886 | 0.792867290814627 |
| MTND1P32 | -0,620162828 | 0,02911149 | -0.0273698641380363 | NA |
| CHRM3 | -0,620020432 | 0,0001896 | -0.150101194323399 | 0.720408284537327 |
| ZNF669 | -0,619183324 | 0,01326263 | 0.0114850599337178 | 0.988970093740796 |
| NAGK | -0,619049345 | 0,00020423 | -0.0456470518542366 | 0.922280899513029 |
| ZNF805 | -0,618816809 | 0,00065271 | -0.168857975882419 | 0.707429185039409 |
| ZNF470 | -0,618641454 | 0,00668671 | -0.136423676700408 | 0.80159766903889 |
| ZFX | -0,618174278 | 0,00025157 | 0.0206440729066668 | 0.967312188621558 |
| CCDC82 | -0,617822913 | 0,00055667 | -0.167112625865254 | 0.706115914054744 |
| REST | -0,617064063 | 0,0001187 | -0.0319669357332692 | 0.945235267820257 |
| ZNF845 | -0,616989857 | 0,00136869 | -0.257312265963563 | 0.574570114296114 |
| RHOQP1 | -0,616791887 | 0,03560322 | -0.0764498513573896 | 0.917451478220486 |
| RGS17 | -0,615562397 | 0,00090583 | -0.209777746972992 | 0.639523521324996 |
| FBXW7 | -0,614582075 | 0,00046562 | 0.0213303810254208 | 0.967312188621558 |
| LINC01980 | -0,614465818 | 0,01331345 | -0.269104622552426 | 0.641113217579193 |
| WHAMMP2 | -0,614447372 | 0,0163127 | -0.138578116982622 | 0.816297831562396 |
| GORAB | -0,614313072 | 0,00228629 | -0.0711644786420054 | 0.893736508705282 |
| MMS22L | -0,614094082 | 0,00035495 | -0.148403679792454 | 0.729370097177694 |
| MBD5 | -0,614067406 | 0,00019074 | -0.23318909170733 | 0.558207621934153 |
| BMF | -0,61362912 | 0,00493335 | -0.351958314254831 | 0.491684051932336 |
| LINC00324 | -0,612507291 | 0,04035832 | -0.088459444317422 | NA |
| DDX17 | -0,612334757 | 0,00030514 | -0.0932978691855861 | 0.821442021544039 |
| DNAL1 | -0,611847597 | 0,00319359 | 0.154473672472742 | 0.762496207273287 |
| TRAPPC13 | -0,611602059 | 0,00112209 | -0.315389668797224 | 0.479371540589469 |
| ZNF766 | -0,610075434 | 0,00143379 | 0.0514912339499784 | 0.922250983699118 |
| STAM-AS1 | -0,608987863 | 0,04206429 | 0.127458203951541 | 0.858603478525429 |
| CMTR2 | -0,608673914 | 0,00252886 | -0.00179081153951145 | 0.998492724661929 |
| ELF1 | -0,608315711 | 0,00044439 | -0.107756283023038 | 0.798677390913918 |
| MXD1 | -0,606625649 | 0,0107398 | 0.197283480282527 | 0.730279081598507 |
| LINC01036 | -0,606171066 | 0,01146659 | -0.331843494686711 | 0.544782901878924 |
| NHLRC3 | -0,60455914 | 0,0040701 | -0.00733272827876984 | 0.990985530297254 |
| PIH1D2 | -0,60391643 | 0,03256412 | -0.0219985609822942 | 0.978158811587227 |
| GNGT1 | -0,602650857 | 0,03972092 | 0.183616139938176 | NA |
| ZNF646 | -0,602190293 | 0,000762 | 0.0141623855719577 | 0.979990112793121 |
| DNAJC27 | -0,602125422 | 0,00812807 | -0.122854076339338 | 0.820390591926934 |
| ZNF614 | -0,601341216 | 0,00060496 | -0.0605728886747524 | 0.898097057960018 |
| PDIK1L | -0,600024504 | 0,01043441 | -0.0541771568948465 | 0.932594710459121 |
| TAS2R15P | -0,599448755 | 0,04390331 | -0.47166029095956 | 0.478875244986783 |
| ZNF585B | -0,5989892 | 0,00063826 | 0.121580281352036 | 0.780343483517128 |
| H2BC6 | -0,598242225 | 0,04078209 | 0.329833223939928 | 0.619605742211831 |
| FSIP2-AS1 | -0,597768188 | 0,04498148 | -0.306533970098952 | NA |
| BBIP1 | -0,597073789 | 0,00468336 | -0.287462269538431 | 0.55949970174492 |
| DUSP10 | -0,597008024 | 0,02176534 | -0.204958506417236 | 0.738841836541156 |
| RBL1 | -0,59453818 | 0,00013466 | 0.047498544506758 | 0.911212382028329 |
| RAB18 | -0,594399999 | 0,00034463 | -0.179596782410064 | 0.661057651071668 |
| RHEBL1 | -0,594335506 | 0,04833145 | 0.151494271700618 | NA |
| ZFYVE1 | -0,593839718 | 0,01277662 | -0.0821613654106365 | 0.893104427920058 |
| SEC31B | -0,593496688 | 0,00385248 | -0.301031267902854 | 0.532005790448031 |
| ABHD16A | -0,593226638 | 0,04488332 | -0.0113996628158403 | NA |
| IPO5P1 | -0,592166384 | 0,00449406 | -0.184396832688202 | 0.715113621517318 |
| MAPK10 | -0,59196379 | 0,01482858 | -0.153840629762716 | 0.786978135352613 |
| RPSAP13 | -0,591422352 | 0,04845326 | -0.168380254743373 | NA |
| N4BP2L1 | -0,590177531 | 0,04351868 | 0.0531795707390541 | NA |
| DEPDC1P2 | -0,590134756 | 0,01972235 | -0.33019971953515 | 0.565816724804457 |
| MLH3 | -0,589324383 | 0,02344436 | -0.0331573521592859 | 0.963779286677717 |
| EID2B | -0,588033682 | 0,0199352 | 0.24211345280983 | 0.682484152989639 |
| IFRD1 | -0,586895089 | 0,00163591 | -0.268734709301507 | 0.539070987391179 |
| ZNF175 | -0,586308731 | 0,00080217 | -0.425888923251571 | 0.271460673954571 |
| ZSWIM8 | -0,586298385 | 0,00631403 | 0.0760483002847392 | 0.891877542615742 |
| SNX16 | -0,586163358 | 0,01428771 | -0.19740939527975 | 0.730279081598507 |
| CEP97 | -0,585666635 | 0,00080943 | -0.288110629310514 | 0.489808732429974 |
| DGCR6L | 0,585627116 | 0,00559961 | 0.198571360066721 | 0.68639892894713 |
| SHANK1 | 0,58669417 | 0,0395762 | -0.153757435083762 | 0.806000736041635 |
| METRNL | 0,586802302 | 0,00595061 | 0.0970485357007575 | 0.849471245186427 |
| SRGAP3 | 0,586922171 | 0,00237659 | 0.257888307474889 | 0.55949970174492 |
| FGFRL1 | 0,587170458 | 0,00063765 | 0.130442444663192 | 0.755373919614963 |
| LAGE3 | 0,587682416 | 0,0060029 | 0.455772641620335 | 0.34023434257615 |
| TSPAN4 | 0,588013292 | 0,00330046 | 0.135001239278846 | 0.770194338222492 |
| COL6A1 | 0,588336319 | 0,0006363 | -0.0191061738510457 | 0.968966021477719 |
| SYT6 | 0,588575558 | 0,02479356 | 0.0304412016209788 | 0.965418374907633 |
| PRDX2 | 0,58890389 | 0,00022353 | 0.216477608557897 | 0.570574206928719 |
| RPL7AP6 | 0,589494272 | 0,00375152 | 0.276037062146898 | 0.556238012638639 |
| HSPBP1 | 0,589767261 | 0,00044589 | 0.399857181156708 | 0.271460673954571 |
| ATP5F1D | 0,590637486 | 0,00200192 | 0.335363657942361 | 0.461670655467635 |
| CD82 | 0,590917439 | 0,03970164 | 0.164125174172603 | NA |
| AGPAT2 | 0,591004336 | 0,0033363 | 0.187233699915367 | 0.693493568925943 |
| STARD10 | 0,591586242 | 0,006016 | 0.248409679562633 | 0.614443528779505 |
| METTL7B | 0,592308829 | 0,02172295 | 0.402688605137563 | 0.485349090401821 |
| PELI3 | 0,592695882 | 0,00625348 | 0.2668490173688 | 0.585833456666483 |
| GPR108 | 0,592958778 | 0,00016288 | 0.216539681448745 | 0.556238012638639 |
| PTMS | 0,593488741 | 0,0033675 | 0.125667007763589 | 0.7923388973596 |
| NKX1-2 | 0,594058201 | 0,0101572 | 0.341229644517755 | 0.511339184989879 |
| CSRP1 | 0,594424369 | 0,04784141 | 0.0964031541069351 | NA |
| TESC | 0,595516329 | 0,01639379 | 0.137390498559019 | 0.806000736041635 |
| SCN4A | 0,595748314 | 0,03579871 | 0.000158772919607142 | NA |
| ISYNA1 | 0,596615668 | 0,00068693 | 0.118249210147992 | 0.778559327989883 |
| OTOG | 0,597189847 | 0,04406384 | 0.00865215276320268 | 0.992189480104869 |
| TWF2 | 0,597194492 | 0,00367165 | 0.428565955637957 | 0.357194886114202 |
| SLC43A1 | 0,59726342 | 0,0006363 | 0.142975520422949 | 0.734148719086826 |
| SNED1 | 0,59748944 | 0,02236324 | 0.164849488455355 | 0.778706237296349 |
| MYH14 | 0,597572209 | 0,00043054 | 0.249147839888249 | 0.532333069575245 |
| KCNG1 | 0,597659692 | 0,00075599 | 0.294544179560212 | 0.478875244986783 |
| BLOC1S1 | 0,597973327 | 0,0296974 | 0.116104083337325 | 0.85745655427041 |
| CATSPERG | 0,598117075 | 0,03324097 | 0.243978287948258 | 0.702459860961799 |
| SYNPO | 0,598374823 | 0,04179965 | -0.149589062328921 | NA |
| ODC1-DT | 0,598414194 | 0,02744168 | 0.741366211743908 | 0.145727266169228 |
| IGFBP2 | 0,599428468 | 0,00098998 | -0.0388830810947612 | 0.937084108231163 |
| OGDHL | 0,600279065 | 0,00191626 | 0.183927964669117 | 0.681940153625886 |
| H1-2 | 0,601659802 | 0,00293518 | -0.0829304801518879 | 0.874360323486989 |
| IGSF8 | 0,603014483 | 0,00868558 | 0.129127450549756 | 0.805550100331031 |
| SLC7A8 | 0,603248532 | 0,00403285 | -0.0767364567132837 | 0.883348440499811 |
| COL9A3 | 0,603498753 | 0,03501707 | 0.230588572512621 | 0.725337767387978 |
| CFD | 0,603530245 | 0,0323012 | 0.330527110845142 | 0.602309776570834 |
| GSN | 0,603850484 | 0,00441186 | 0.123277570279867 | 0.800751550741699 |
| GRIN2D | 0,603943269 | 0,00207092 | 0.300808610163282 | 0.500987778813949 |
| ENDOG | 0,60428243 | 0,00063802 | 0.374369993414671 | 0.357194886114202 |
| RAB3D | 0,60430704 | 0,0007712 | 0.139449779169983 | 0.748934067099268 |
| FGFR4 | 0,606050181 | 0,00019154 | 0.189822938800173 | 0.620642813223097 |
| NQO1 | 0,606094465 | 0,04160497 | 0.302006146799576 | NA |
| CDH1 | 0,606379404 | 0,02064538 | -0.0116353141920587 | 0.988657125700466 |
| GAPDHP1 | 0,606600603 | 0,00994052 | 0.297401954524437 | 0.582558442417588 |
| KCNN1 | 0,607172089 | 0,03264211 | -0.0882171417390178 | 0.899217512293142 |
| MAFA | 0,607857093 | 0,03419437 | 0.598834258606241 | NA |
| NMB | 0,608287094 | 0,01423546 | 0.0869672731789049 | 0.88653652546864 |
| CRAT | 0,608729248 | 0,0120633 | 0.228880051130653 | 0.682078016759553 |
| SOX8 | 0,609720845 | 0,00824763 | 0.22046013161365 | 0.676831002778033 |
| C16orf74 | 0,609870282 | 0,03443693 | 0.132813137771572 | 0.843616802502219 |
| NGEF | 0,609884411 | 0,00982131 | 0.0632144678618073 | 0.91427397178527 |
| SLC6A16 | 0,609996938 | 0,02064538 | -0.0728476257948803 | 0.911269644302293 |
| PLIN1 | 0,610422722 | 0,03624008 | 0.36616882303323 | NA |
| SCAND1 | 0,610785 | 0,00394424 | 0.546362530880395 | 0.206335521108117 |
| UCP2 | 0,611294646 | 0,00068363 | 0.229382732897293 | 0.587383877141507 |
| USH1C | 0,611891336 | 0,01577827 | 0.0126407509976849 | 0.987366825714395 |
| CITED4 | 0,612016221 | 0,0405557 | 0.432443984588536 | NA |
| COL2A1 | 0,612079402 | 0,00087813 | 0.145244087084965 | 0.738484850420232 |
| PPP1R16B | 0,612291003 | 0,01836372 | -0.122883454019888 | 0.836972114703054 |
| AGTRAP | 0,614727685 | 0,00033142 | 0.278209038960372 | 0.484994778033328 |
| RPS2P55 | 0,615332869 | 0,03324191 | 0.0586753130864285 | NA |
| TMEM37 | 0,615368312 | 0,02548939 | 0.0799921669884151 | 0.904712451638182 |
| ASS1 | 0,615746859 | 0,00094547 | 0.00251037043232815 | 0.997223944630306 |
| DENND1C | 0,616324548 | 0,03910516 | 0.000855514244584341 | NA |
| BDH1 | 0,616384064 | 0,03806311 | 0.135357330763375 | NA |
| RPL5P23 | 0,616395725 | 0,00366459 | 0.429309694786065 | 0.368861955384111 |
| PC | 0,61651656 | 0,00041101 | 0.480834226624722 | 0.181985129780982 |
| ITGA2B | 0,617214122 | 0,02064538 | 0.0719931318435208 | 0.913486735748136 |
| H4C3 | 0,617393026 | 0,00297838 | 0.300027174556173 | 0.538759045740695 |
| OLFML2A | 0,617705822 | 0,03137511 | -0.0321532382559206 | NA |
| PRXL2B | 0,617737208 | 0,02609668 | 0.118881821581604 | 0.857075277811286 |
| TPST2 | 0,618014634 | 0,00045094 | 0.288034816393811 | 0.48157975071739 |
| ABHD14A | 0,618349719 | 0,02978502 | 0.318274450235728 | NA |
| PLCD1 | 0,618789568 | 0,02520133 | -0.00295924614689168 | 0.997767140349553 |
| MAST1 | 0,619320383 | 0,00451575 | -0.116470509837235 | 0.813862921340934 |
| EIF4BP7 | 0,619950517 | 0,00628595 | 0.153451703656931 | 0.770145332677736 |
| JOSD2 | 0,620541778 | 0,01283091 | 0.493819321017705 | 0.369781661436704 |
| SLC41A3 | 0,62129597 | 0,00020963 | -0.05417071464486 | 0.900021856081565 |
| NYNRIN | 0,622280335 | 0,00608258 | 0.0007686036266159 | 0.999666741877378 |
| KCNAB2 | 0,622378612 | 0,01061538 | 0.0498215064897963 | 0.93882867950986 |
| S1PR2 | 0,622703108 | 0,00078138 | 0.360250031672046 | 0.397981934438104 |
| BTD | 0,622924447 | 0,0061809 | 0.0797859309365691 | 0.890740389753551 |
| PEMT | 0,624828146 | 0,00011238 | 0.261489842471224 | 0.491684051932336 |
| TMEM205 | 0,624971546 | 0,00090075 | 0.117725552179173 | 0.787269269420425 |
| KREMEN2 | 0,625181941 | 0,0296974 | 0.44607230999034 | NA |
| SNTG2 | 0,625222529 | 0,02814176 | 0.471671803486685 | NA |
| SYT3 | 0,625409465 | 0,03207147 | 0.15850653787097 | NA |
| ECEL1 | 0,625431099 | 0,01404965 | 0.0903519918027031 | 0.885990769160661 |
| RGS3 | 0,626508176 | 0,03488021 | 0.383706915544152 | NA |
| RTN2 | 0,626763486 | 0,01281087 | 0.0484839032755785 | 0.943750215029897 |
| DACT3 | 0,627012689 | 0,02987783 | 0.343011077685772 | NA |
| RTN4RL1 | 0,629112734 | 0,00527959 | 0.00785291964385287 | 0.990920312802863 |
| MMP15 | 0,629120038 | 0,00373903 | 0.0670284222721728 | 0.901911166522359 |
| KCNQ2 | 0,62976417 | 0,00024741 | 0.253838231393014 | 0.531984735417389 |
| SLC8B1 | 0,629839177 | 0,0060029 | 0.0897977387275623 | 0.873861331320875 |
| GDPD5 | 0,629844587 | 0,00407454 | -0.0130389336739251 | 0.984097128323686 |
| EFHD1 | 0,630509564 | 0,00046562 | 0.0770271757009265 | 0.865363272614317 |
| NPTX2 | 0,632462643 | 0,00367165 | 0.0502088245873684 | 0.929832823153942 |
| MYOM2 | 0,632560854 | 0,00286789 | 0.099145455690351 | 0.846804425819919 |
| S100A11 | 0,634452074 | 0,00128911 | 0.115950394892467 | 0.803350565192274 |
| CSPG4 | 0,634499394 | 0,01651945 | 0.247825080226825 | 0.680418040657311 |
| RNF208 | 0,634706143 | 0,0096918 | 0.472601824429674 | 0.385883386322168 |
| ASIC3 | 0,634717581 | 0,01344423 | 0.0891864875851051 | 0.887751829234438 |
| SSPOP | 0,635108957 | 0,02237894 | 0.0922768999516918 | 0.893552912845511 |
| ZCCHC24 | 0,635855188 | 0,00194042 | -0.0217565772814318 | 0.969314345431145 |
| KCNJ4 | 0,636156297 | 0,03204349 | 0.267089110893985 | NA |
| TBXAS1 | 0,637491078 | 0,0100067 | 0.283687614150534 | 0.614443528779505 |
| FCHO1 | 0,637708641 | 0,01939371 | 0.138272036840379 | 0.822857126073835 |
| PROCR | 0,638001653 | 0,00705148 | -0.168219481340439 | 0.761220780847536 |
| FOXO6 | 0,640382807 | 0,02986713 | 0.036502405729491 | NA |
| STUM | 0,641218092 | 0,00469002 | -0.102527207946028 | 0.849471245186427 |
| MFSD10 | 0,642016442 | 0,00017027 | 0.391514278824292 | 0.297570971715301 |
| H2AC11 | 0,643217209 | 0,00102853 | -0.00412340850561259 | 0.994889455578659 |
| THEM6 | 0,643379225 | 0,00652703 | 0.267941600082825 | 0.62105875807683 |
| RTN3P1 | 0,644582084 | 0,02015649 | 0.190625121464147 | 0.766283264049794 |
| LRRC27 | 0,645750876 | 0,01363655 | 0.484238170598383 | 0.413767975247101 |
| SLC9A3R2 | 0,645933487 | 0,00090831 | 0.244319908771569 | 0.594068965537461 |
| TMEM53 | 0,646409461 | 0,01433524 | 0.305210373245995 | 0.609913168713549 |
| CAMK2B | 0,646684165 | 0,02359326 | 0.52077661636937 | NA |
| GRID2IP | 0,64674032 | 0,01746754 | 0.131571957643158 | 0.833121652864346 |
| RPL13P12 | 0,647683118 | 0,00449488 | -0.0625064231845618 | 0.914053854869602 |
| ALPL | 0,648564208 | 0,01954822 | 0.0585203011599775 | 0.93625269720115 |
| B4GALNT1 | 0,648716645 | 0,00087375 | -0.0534167276821543 | 0.901911166522359 |
| RHBDF2 | 0,649916889 | 0,00036473 | 0.38407821107188 | 0.357194886114202 |
| TMEM266 | 0,649928513 | 0,00898654 | -0.0412760619148838 | 0.951437144281309 |
| LINC01232 | 0,651954157 | 0,02529271 | 0.635754373498391 | NA |
| SYNGR1 | 0,653240944 | 0,00018068 | 0.203691667632339 | 0.623668552821515 |
| POLR2J4 | 0,655293444 | 0,02311274 | 0.403950352333691 | NA |
| ARHGEF17 | 0,657113478 | 0,00180905 | 0.121487742384811 | 0.803172722801883 |
| ZNF775 | 0,657166295 | 0,00672813 | 0.277757951821041 | 0.61808529801756 |
| CNN2 | 0,657480316 | 0,00023961 | 0.178019015600386 | 0.677174537526981 |
| PPDPF | 0,657560859 | 0,00242266 | 0.275527952934372 | 0.586899823639234 |
| LMOD1 | 0,657784308 | 0,01248126 | 0.228178133166161 | 0.711422360713909 |
| SPR | 0,658255235 | 0,00415491 | 0.402723215851641 | 0.455663658287657 |
| FLVCR2 | 0,658549524 | 0,02154622 | 0.0320449372991723 | NA |
| CTNNBIP1 | 0,658621723 | 0,00286496 | 0.148756764877595 | 0.769544264631444 |
| TYSND1 | 0,66020577 | 0,00063623 | 0.433411512054742 | 0.306299263486855 |
| ZNF358 | 0,660374687 | 0,00011436 | 0.301871566832106 | 0.460079856082012 |
| HSPG2 | 0,663302927 | 0,00165443 | 0.0446543036764665 | 0.937890479656424 |
| H4C2 | 0,664363933 | 0,00196519 | 0.29553426867208 | 0.558207621934153 |
| CRIP2 | 0,664968203 | 0,01631548 | 0.138178138155184 | 0.825222114056771 |
| RETREG1 | 0,665233687 | 0,0090032 | 0.293264718602124 | 0.614518167181335 |
| HBQ1 | 0,665524263 | 0,02368324 | -0.176777879447301 | NA |
| IGDCC4 | 0,665619628 | 0,00046847 | 0.131885303776118 | 0.768398476264955 |
| ABCC6 | 0,666274457 | 0,01192353 | 0.373514932493091 | 0.532333069575245 |
| FTH1P7 | 0,666423782 | 0,01180796 | 0.65739739866824 | 0.234451104549597 |
| ADGRA2 | 0,667056525 | 0,00067476 |  |  |
| CTXN1 | 0,668001778 | 0,0096286 | 0.419514401728204 | 0.479224215643066 |
| AHNAK2 | 0,671361695 | 0,00052646 | -0.0812359832069932 | 0.867570620584379 |
| SLIT1 | 0,671824953 | 0,00699659 | 0.128918928660428 | 0.821098199219315 |
| EGFL7 | 0,67189654 | 0,00228161 | 0.3632312741656 | 0.476438741954515 |
| PLTP | 0,673627625 | 0,00044856 | 0.149679337789535 | 0.747865830624954 |
| TRPM4 | 0,674900175 | 0,00222333 | 0.0698340235732099 | 0.901231796012132 |
| CACNA1B | 0,675022791 | 0,00232001 | 0.29156479773042 | 0.562415904695673 |
| H1-3 | 0,675381925 | 0,0001896 | -0.050388864368649 | 0.917451478220486 |
| ZNF517 | 0,675972804 | 0,00766557 | 0.252299918239925 | 0.664837857230756 |
| ACSS2 | 0,676153195 | 0,00163444 | 0.0177087929854326 | 0.976578928943165 |
| SLC2A6 | 0,6781404 | 0,00424241 | 0.280299122291827 | 0.612292241984978 |
| WNT3A | 0,678177476 | 0,00315209 | 0.232124004544718 | 0.662063583153021 |
| ABCD1 | 0,678446897 | 0,00582476 | 0.139390491556158 | 0.804718054660669 |
| SLC29A4 | 0,679762865 | 0,0013232 | -0.0840394922195059 | 0.874360323486989 |
| SEMA3G | 0,679767496 | 0,00617862 | 0.343714630164401 | 0.538759045740695 |
| TGM2 | 0,680461794 | 0,01664831 | -0.134939721729371 | NA |
| FAM78A | 0,680735272 | 0,00403285 | 0.0508156815143005 | 0.936364654306675 |
| IGDCC3 | 0,681569488 | 0,00087375 | 0.18363893328926 | 0.707772022514914 |
| METRN | 0,681880747 | 0,00453879 | 0.0742310740590565 | 0.901911166522359 |
| OCA2 | 0,683537792 | 0,00792768 | -0.0192955308180489 | 0.979735576695182 |
| DPP7 | 0,684421736 | 0,01110462 | 0.793817016318433 | 0.0975806225368274 |
| ELFN1 | 0,684650341 | 0,00183975 | 0.18514616171041 | 0.722226440872635 |
| CDPF1 | 0,685645618 | 0,00296364 | 0.287639621078917 | 0.584082174278205 |
| H3C10 | 0,685803436 | 0,00029223 | 0.142081741119656 | 0.763706670294856 |
| LINC00863 | 0,686423209 | 0,0058027 | 0.41469933551417 | 0.47355214335331 |
| SIGIRR | 0,688848159 | 0,00235973 | 0.253959407164105 | 0.629435476691396 |
| CD27-AS1 | 0,689990661 | 0,00701372 | 0.192662665042926 | 0.747493386702251 |
| CLEC11A | 0,690584816 | 0,01067962 | 0.464350476211865 | 0.45796535096302 |
| ATP2A3 | 0,692912449 | 0,00602806 | 0.0140941063629853 | 0.986291159669459 |
| H4-16 | 0,693138336 | 0,00046847 | 0.0496748017941152 | 0.927942609334684 |
| PRRT4 | 0,694960734 | 0,00181565 | 0.332448682557414 | 0.514948163136529 |
| COMP | 0,697361032 | 0,0129467 | 0.404635884574158 | 0.528352654342201 |
| RPS26P6 | 0,697431164 | 0,01435585 | 0.478528494666294 | NA |
| H3C15 | 0,704319557 | 0,0137317 | 0.409445387434211 | 0.531984735417389 |
| RRAS | 0,705704325 | 0,01225355 | 0.213539047753493 | 0.747765785719127 |
| C9orf16 | 0,705964759 | 0,00246875 | 0.278293126208422 | 0.613133534845239 |
| EEF1B2P6 | 0,706712188 | 0,01343588 | 0.0857263775898437 | 0.904712451638182 |
| RHPN1 | 0,710753874 | 0,00080943 | 0.298265587436121 | 0.538759045740695 |
| ZDHHC24 | 0,711094415 | 0,00014794 |  |  |
| STN1 | 0,711371387 | 0,00152119 | 0.108986588730417 | 0.8375618039879 |
| C1QL1 | 0,718257841 | 0,00668671 | -0.0147622639535092 | 0.986291159669459 |
| TLL2 | 0,721131144 | 0,01231073 | 0.12368659456887 | NA |
| WNT11 | 0,721692498 | 0,00675159 | 0.16934356401354 | 0.7834903552204 |
| ALKBH7 | 0,726047889 | 0,0004518 | 0.242201648940715 | 0.61631722935403 |
| FAM86FP | 0,726304993 | 0,00763147 | 0.196897073746551 | 0.702459860961799 |
| MIR210HG | 0,72748165 | 0,01162822 | 0.0552896112657482 | NA |
| PLPPR3 | 0,728766441 | 0,00652334 | 0.0407837742695581 | 0.955678923845787 |
| H3C2 | 0,731561034 | 0,00011902 | 0.123164977263797 | 0.792320224663542 |
| LRRK1 | 0,732184546 | 0,00699688 | 0.110273991205312 | 0.870028403137167 |
| RANBP1P1 | 0,733678896 | 0,0056681 | -0.112933593497943 | NA |
| EPHA8 | 0,736510548 | 0,0016184 | 0.541762414915166 | 0.280090761826511 |
| RASSF4 | 0,740190384 | 0,00451052 | -0.24175132455512 | 0.680418040657311 |
| NPAS1 | 0,741506684 | 0,0001896 | 0.245122658750334 | 0.605416958494931 |
| F12 | 0,7427942 | 0,00146559 | 0.416205044842059 | 0.448446237360309 |
| PANX2 | 0,743916251 | 0,0003908 | 0.200439720505256 | 0.68622049655762 |
| MYO15B | 0,749451516 | 0,00484418 | 0.0307190756841293 | 0.967312188621558 |
| HDAC11 | 0,750871853 | 0,00132931 | 0.192489280303546 | 0.726258443269629 |
| H2AC21 | 0,752467031 | 0,00026177 | 0.256138584530428 | 0.607419238022118 |
| CCDC3 | 0,756528619 | 0,00016212 | 0.121683255310658 | 0.793683364210634 |
| ATP1B2 | 0,763146777 | 0,00065271 | -0.0755384275143575 | 0.894140746840539 |
| TUBB4A | 0,769887475 | 0,00059526 | 0.151420719320149 | NA |
| B3GNT4 | 0,774186567 | 0,00334169 | 0.126310770495367 | 0.840131641437003 |
| STAC2 | 0,776588995 | 0,00193752 | 0.0706604938104231 | 0.912413611352967 |
| RPS3AP6 | 0,786205585 | 0,0028156 | 0.521424782746244 | 0.385883386322168 |
| RAB3A | 0,792565222 | 0,00103169 | 0.269987125978436 | 0.631916368535332 |
| TNFAIP8L1 | 0,798530023 | 0,0015713 | 0.188846321782484 | 0.751794385299391 |
| H2AC15 | 0,799193601 | 0,00015364 | 0.222704310571248 | 0.664730642994791 |
| KHK | 0,802568328 | 0,00090075 | 0.213194574829384 | 0.711506418520065 |
| PALM | 0,826519277 | 0,00016531 | 0.223913520196903 | 0.6744217336548 |
| PIK3CD | 0,830086112 | 0,00114825 | 0.114653216525463 | 0.854119595189263 |
| REEP6 | 0,834794202 | 0,00029223 | 0.237418132874566 | 0.667325344599479 |
| RPS2P7 | 0,841667781 | 0,00011362 | 0.439073083560595 | 0.401954641961234 |
| RPSAP15 | 0,853766031 | 0,00010747 | 0.486097568718174 | 0.34023434257615 |
| SCUBE1 | 0,860077753 | 0,0004835 | 0.0187241281935374 | 0.97995337862636 |
| H2AC19 | 0,865248092 | 0,00043949 | 0.422962340893301 | 0.476438741954515 |
| ATP1A3 | 0,887097855 | 0,00164345 | 0.338140783041044 | NA |
| TRIM7 | 0,901362411 | 0,00120154 | 0.161192296401747 | NA |
| H4C4 | 0,905140805 | 0,00028875 | 0.205640456600763 | 0.740061224075768 |
| RTN4R | 0,91341807 | 0,00043689 | 0.431518406609993 | 0.478875244986783 |
