# Supplementary material for: Stable structures or PABP1 loading protects cellular and viral RNAs against ISG20-mediated decay
Source: Life Sci Alliance. 2024 Feb 28;7(5):e202302233. doi: 10.26508/lsa.202302233 (PMC10902665; doi:10.26508/lsa.202302233)
Supplement: Supplementary file 4 [file LSA-2023-02233_TableS2.docx]

**Supplementary Table 2.**

| **IGR-L** **structural element** | **δG** | **Information** |
| --- | --- | --- |
| External loop | 0.00 | 0 ss bases & 1 closing helices |
| Stack | -3.30 | External closing pair is C1-G39 |
| Stack | -3.30 | External closing pair is C2-G38 |
| Stack | -3.30 | External closing pair is C3-G37 |
| Stack | -3.30 | External closing pair is C4-G36 |
| Stack | -2.40 | External closing pair is C5-G35 |
| Stack | -2.40 | External closing pair is G6-C34 |
| Stack | -2.10 | External closing pair is A7-U33 |
| Stack | -2.40 | External closing pair is G8-C32 |
| Stack | -2.20 | External closing pair is A9-U31 |
| Stack | -3.30 | External closing pair is C10-G30 |
| Stack | -3.30 | External closing pair is C11-G29 |
| Stack | -2.10 | External closing pair is C12-G28 |
| Stack | -2.20 | External closing pair is A13-U27 |
| Stack | -3.30 | External closing pair is C14-C26 |
| Stack | -2.40 | External closing pair is C15-G25 |
| Stack | -3.40 | External closing pair is G16-C24 |
| Stack | -3.30 | External closing pair is C17-G23 |
| **Helix** | -48.00 | 18 base pairs |
| Hairpin loop | 5.70 | External closing pair is C18-G22 |
| **ΔG= -42.30 kca/mol** |  |  |
| **IGR-S** **structural element** | **δG** | **Information** |
| External loop | -0.30 | 1 ss bases & 1 closing helices |
| Stack | -3.30 | External closing pair is C2-G39 |
| Stack | -3.30 | External closing pair is C3-G38 |
| Stack | -3.30 | External closing pair is C4-G37 |
| Stack | -3.30 | External closing pair is C5-G36 |
| Stack | -2.40 | External closing pair is C6-G35 |
| Stack | -3.30 | External closing pair is G7-C34 |
| Stack | -3.30 | External closing pair is G8-C33 |
| Stack | -3.30 | External closing pair is G9-C32 |
| Stack | -3.30 | External closing pair is G10-C31 |
| Stack | -3.30 | External closing pair is G11-C30 |
| Stack | -2.40 | External closing pair is G12-C29 |
| **Helix** | -34.50 | 12 base pairs |
| Interior loop | -1.00 | External closing pair is A13-U28 |
| Stack | -3.30 | External closing pair is C15-G26 |
| Stack | -3.30 | External closing pair is C16-G25 |
| Stack | -3.30 | External closing pair is C17-G24 |
| Helix | -9.90 | 4 base pairs |
| Hairpin loop | 4.00 | Closing pair is C18-G23 |
| **ΔG= -42.30 kca/mol** |  |  |
