# Supplementary material for: Stable structures or PABP1 loading protects cellular and viral RNAs against ISG20-mediated decay
Source: Life Sci Alliance. 2024 Feb 28;7(5):e202302233. doi: 10.26508/lsa.202302233 (PMC10902665; doi:10.26508/lsa.202302233)
Supplement: Supplementary file 5 [file LSA-2023-02233_TableS3.docx]

**Supplementary Table 3.**

| **Histone** **structural element** | **δG** | **Information** |
| --- | --- | --- |
| External loop | 0.00 | 0 ss bases & 1 closing helix |
| Stack | -3.30 | External closing pair is G1-C16 |
| Stack | -3.40 | External closing pair is G2-C15 |
| Stack | -2.10 | External closing pair is C3-G14 |
| Stack | -2.40 | External closing pair is U4-A13 |
| Stack | -2.10 | External closing pair is C5-G12 |
| **Helix** | -13.30 | 6 base pairs |
| Hairpin loop | 5.50 | Closing pair is U6-A11 |
| **ΔG= -7.80 kca/mol** |  |  |
