# Supplementary material for: Stable structures or PABP1 loading protects cellular and viral RNAs against ISG20-mediated decay
Source: Life Sci Alliance. 2024 Feb 28;7(5):e202302233. doi: 10.26508/lsa.202302233 (PMC10902665; doi:10.26508/lsa.202302233)
Supplement: Supplementary file 6 [file LSA-2023-02233_TableS4.docx]

**Supplementary Table 4.**

| **TAR** **structural element** | **δG** | **Information** |
| --- | --- | --- |
| External loop | 0.00 | 0 ss bases & 1 closing helices |
| Stack | -3.30 | External closing pair is G1-C59 |
| Stack | -3.30 | External closing pair is G2- C58 |
| Stack | -2.20 | External closing pair is G3-C57 |
| **Helix** | -8.80 | 4 base pairs |
| **Bulge loop** | 2.90 | External closing pair is U4-C56 |
| Stack | -2.40 | External closing pair is U6-A55 |
| Stack | -2.10 | External closing pair is C7-G54 |
| Stack | -1.50 | External closing pair is U8-G53 |
| Stack | -2.10 | External closing pair is C9-G52 |
| Stack | -1.00 | External closing pair is U10-A51 |
| Stack | -2.10 | External closing pair is G11-U50 |
| Stack | -2.20 | External closing pair is G12-C49 |
| Stack | -0.90 | External closing pair is U13-A48 |
| Stack | -1.30 | External closing pair is U14-A47 |
| Stack | -2.10 | External closing pair is A15-U46 |
| **Helix** | -17.70 | 11 base pairs |
| **Bulge loop** | 0.40 | External closing pair is G16-C45 |
| Stack | -3.30 | External closing pair is C18-G44 |
| Stack | -2.10 | External closing pair is C19-G43 |
| Stack | -2.10 | External closing pair is A20-U42 |
| Stack | -2.40 | External closing pair is G21-C41 |
| **Helix** | -9.90 | 5 base pairs |
| **Bulge loop** | 3.70 | External closing pair is A22-U40 |
| Stack | -2.40 | External closing pair is G26-C39 |
| Stack | -2.10 | External closing pair is A27-U38 |
| Stack | -3.40 | External closing pair is G28-C37 |
| **Helix** | -7.90 | 4 base pairs |
| Hairpin loop | 4.40 | Closing pair is C29-G36 |
| **ΔG= -32.90 kca/mol** |  |  |

**Supplementary Table 4, continued.**

| **TAR-5SL** **structural element** | **δG** | **Information** |
| --- | --- | --- |
| External loop | 0.00 | 0 ss bases & 1 closing helix |
| Stack | -3.40 | External closing pair is G1-C27 |
| Stack | -2.10 | External closing pair is C2- G26 |
| Stack | -2.10 | External closing pair is A3-U25 |
| Stack | -2.40 | External closing pair is G4-C24 |
| **Helix** | -10.00 | 5 base pairs |
| Bulge loop | -3.70 | External closing pair is A5-U23 |
| Stack | -2.40 | External closing pair is G9-C22 |
| Stack | -2.10 | External closing pair is A10-U21 |
| Stack | -3.40 | External closing pair is G11-C20 |
| **Helix** | -7.90 | External closing pair is 4 base pairs |
| Hairpin loop | 4.40 | External closing pair is C12-G19 |
| **ΔG= -9.80 kcal/mol** |  |  |
| **TAR-9SL structural element** | **δG** | **Information** |
| External loop | 0.00 | 0 ss bases & 1 closing helix |
| Stack | -3.30 | External closing pair is G1-C35 |
| Stack | -3.30 | External closing pair is G2- C34 |
| Stack | -3.30 | External closing pair is G3-C33 |
| Stack | -3.40 | External closing pair is G4-C32 |
| Stack | -3.30 | External closing pair is C5-G31 |
| Stack | -2.10 | External closing pair is C6-G30 |
| Stack | -2.10 | External closing pair is A7-U29 |
| Stack | -2.40 | External closing pair is G8-C29 |
| **Helix** | -23.20 | 9 base pairs |
| Bulge loop | -3.70 | External closing pair is A9-U27 |
| Stack | -2.40 | External closing pair is G13-C26 |
| Stack | -2.10 | External closing pair is A14-U25 |
| Stack | -3.40 | External closing pair is G15-C24 |
| **Helix** | -7.90 | External closing pair is 4 base pairs |
| Hairpin loop | 4.40 | External closing pair is C16-G23 |
| **ΔG= -23.00 kca/mol** |  |  |
