# Supplementary material for: Stable structures or PABP1 loading protects cellular and viral RNAs against ISG20-mediated decay
Source: Life Sci Alliance. 2024 Feb 28;7(5):e202302233. doi: 10.26508/lsa.202302233 (PMC10902665; doi:10.26508/lsa.202302233)
Supplement: Supplementary file 7 [file LSA-2023-02233_TableS5.docx]

**Supplementary Table 5.**

| **Name** | **Sequence (5’- 3’)** |
| --- | --- |
| PT7 | AAATAATACGACTCACTATAGGG |
| FF3 | AAATAATACGACTCACTATAGGGGCCAGATCTGAGCCTGGGAGCTCTCTGGCCCC |
| FF4 | GGGCCAGAGAGCTCCCAGGCTCAGATCTGGTCCCTATAGTGAGTCGTATTATTT |
| FF5 | GGGTTCCCTAGTTAGCCAGAGAGCTCCCAGGCTCAGATCTGGTCTAACCAGAGAGACCCTATAGTGAGTCGTATTATTT |
| MOPV-NP-forward | GTCAAGCGTTCTTTGGGAATG |
| MOPV-NP-reverse | TCCAGAAAGACATAGTTTGTAGAGG |
| MOPV-NP-probe | FAM-TTCCTTTCCCCTGGCGTGTCA-BHQ1 |
