# Supplementary material for: Stable structures or PABP1 loading protects cellular and viral RNAs against ISG20-mediated decay
Source: Life Sci Alliance. 2024 Feb 28;7(5):e202302233. doi: 10.26508/lsa.202302233 (PMC10902665; doi:10.26508/lsa.202302233)
Supplement: Supplementary file 8 [file LSA-2023-02233_TableS6.docx]

**Supplementary Table 6.**

| **Name** | **Sequence (5’- 3’)** | **Synthesis** |
| --- | --- | --- |
| (AC)_10_A_20_ | ACACACACACACACACACACAAAAAAAAAAAAAAAAAAAA | chemically synthesized |
| (AC)_10_ | ACACACACACACACACACAC | chemically synthesized |
| Histone 3’ | GGCUCUUUUCAGAGCC | chemically synthesized |
| MOPV (IGR-L) | CCCCCGAGACCCACCGCCGAAGGCGGUGGGUCUCGGGGG | chemically synthesized |
| TAR-5SL | GCAGAUCUGAGCCUGGGAGCUCUCUGC | chemically synthesized |
| TAR-9SL | GGGGCCAGAUCUGAGCCUGGGAGCUCUCUGGCCC | FF3/FF4 hybridization |
| TAR | GGGUCUCUCUGGUUAGACCAGAUCUGAGCCUGGGAGCUCUCUGGCUAACUAGGGAACCC | PT7/FF5 hybridization |
